# Evaluation of febuxostat in treating diabetic kidney disease with hyperuricemia: a systematic review and meta-analysis of randomized controlled trials

**DOI:** 10.3389/fmed.2025.1657274

**Published:** 2025-10-27

**Authors:** Minghao Lin, Hui Zhang, Haonan Wu, Dexi Zhao, Zheng Nan, Yujuan Fu

**Affiliations:** ^1^Changchun University of Chinese Medicine, Changchun, China; ^2^The Affiliated Hospital to Changchun University of Chinese Medicine, Changchun, China

**Keywords:** febuxostat, hyperuricemia, diabetic kidney disease, meta-analysis, randomized controlled trial

## Abstract

**Background:**

Diabetic kidney disease (DKD) combined with hyperuricemia (HUA) constitutes a pathological state of vicious cycle, where diabetic microvascular complications affect the kidneys and interact with persistent hyperuricemia. This condition significantly accelerates the progression of renal failure and increases all-cause mortality.

**Objective:**

This study aims to systematically evaluate the clinical efficacy and safety of febuxostat in treating patients with DKD and HUA via a meta-analysis, thereby providing evidence-based guidance for optimizing clinical medication.

**Methods:**

Following the PICOS principle, we systematically searched for randomized controlled trials (RCTs) on febuxostat for treating DKD combined with HUA, covering the period from the establishment of each database to June 26, 2025. Studies meeting the inclusion criteria were selected, and a meta-analysis was performed using Review Manager 5.4 software.

**Results:**

A total of 17 RCTs were included, involving 1,300 patients (treatment group n=647, control group *n* = 653). The results of the meta-analysis showed that the overall effective rate of the febuxostat treatment group was significantly higher than that of the control group (RR = 1.24, 95%CI: 1.17–1.32; Z = 7.17, *P* < 0.001). In addition, febuxostat significantly reduced serum uric acid (SUA), urinary albumin-to-creatinine ratio (UACR), serum creatinine (Scr), and blood urea nitrogen (BUN) levels, and improved the estimated glomerular filtration rate (eGFR) (*P* < 0.001 for all indicators).

**Conclusion:**

This meta-analysis indicates that febuxostat, when used to treat patients with DKD and HUA, can significantly enhance overall clinical effectiveness and effectively improve key renal function indicators—including SUA, UACR, Scr, BUN, and eGFR. The results of this study showed that when febuxostat is used to treat patients with diabetic nephropathy complicated by hyperuricemia, it achieves a higher overall clinical response rate. It can significantly reduce the levels of SUA, UACR, Scr, and BUN in patients, while improving the eGFR. Additionally, the incidence of adverse reactions associated with febuxostat is lower, suggesting that this drug exhibits favorable clinical safety.

**Systematic review registration:**

https://www.crd.york.ac.uk/PROSPERO/.

## 1 Introduction

With the continuous rise in the global prevalence of diabetes, DKD has become one of the most severe and common microvascular complications of diabetes and is the leading cause of end-stage renal disease (ESRD) (1). The pathophysiological mechanisms of DKD are complex, involving multiple factors such as hemodynamic changes, metabolic disorders, oxidative stress, inflammatory responses, and fibrosis (2). Effectively delaying the progression of DKD and reducing the risk of ESRD and cardiovascular complications are important challenges currently faced in clinical practice (3).

In recent years, an increasing number of clinical and basic studies have suggested that HUA plays a significant role in the occurrence and development of DKD (4). Elevated blood uric acid levels are not only closely related to insulin resistance and metabolic syndrome and are considered an important risk factor for the development of diabetes; more importantly, uric acid, as an important endogenous “damage-associated molecular pattern” (DAMP), can induce renal tubular epithelial cell damage, activate the local renin-angiotensin system (RAS) in the kidneys, promote oxidative stress and the release of pro-inflammatory factors (such as IL-1β, TNF-α), and exacerbate renal inflammatory infiltration and tissue fibrosis (5). A large amount of epidemiological evidence has shown that elevated serum uric acid levels are an independent risk factor for renal function decline and adverse renal outcomes in patients with DKD. Therefore, actively intervening in HUA may provide a new target for DKD management (6).

Febuxostat, a selective xanthine oxidase inhibitor, has gained increasing clinical application due to its strong efficacy in lowering uric acid levels and better tolerability in patients with impaired renal function compared to allopurinol (especially because of its non-renal-dependent excretion) (7). Basic research has shown that in addition to effectively reducing uric acid, febuxostat also has potential anti-inflammatory, antioxidant, and endothelial function-improving effects. Some clinical studies have observed that febuxostat can slow the decline in renal function and reduce proteinuria in patients with DKD and HUA, suggesting its possible renal-protective effects. However, the existing clinical evidence mainly comes from individual RCTs, and the results are not entirely consistent (8). While some studies have observed significant renal benefits, others have suggested that it is controversial or has no obvious advantages in terms of renal function impact (9). This heterogeneity in study results stems from a variety of factors, including differences in sample size, baseline characteristics of the study subjects (such as DKD stage, baseline uric acid levels, concomitant medications), duration of treatment, and evaluation indicators of therapeutic effects.

In order to evaluate more comprehensively and objectively the exact efficacy and safety of febuxostat treatment for patients with hyperuricemia complicated with diabetic nephropathy, especially its impact on key indicators such as renal function, such as estimated glomerular filtration rate eGFR, serum creatinine, proteinuria such as urinary albumin/creatinine ratio UACR, and adverse renal events, there is an urgent need to conduct systematic integration and quantitative analysis of the existing RCTs (10). To this end, this study plans to strictly screen and include relevant high-quality randomized controlled trials through the methods of “Systematic Review” and “meta-analysis”, aiming to explore the following core issues: ① Efficacy evaluation: To evaluate the efficacy of febuxostat treatment in delaying the deterioration of renal function (eGFR decline rate, creatinine level) in patients with hyperuricemia complicated with diabetic nephropathy compared with placebo or other active control drugs (such as allopurinol); Clarify its effect on reducing proteinuria (UACR). ② Safety considerations: Evaluate the safety and tolerability of febuxostat treatment in this specific population, with a focus on analyzing the incidence of adverse reactions related to deterioration of renal function. ③ Evidence integration: Integrate the best existing evidence and assess its extent of supporting the therapeutic value of febuxostat in the management of such patients. The findings of this study will provide clinicians with a higher-level evidence-based medical basis for formulating individualized uric acid-lowering treatment plans for patients with diabetic nephropathy complicated with hyperuricemia.

## 2 Data and methods

This study has been registered with PROSPERO (International Prospective Register of Systematic Reviews) with the study number CRD420251081616. This meta-analysis was conducted strictly in accordance with the PRISMA guidelines. A comprehensive search was performed in Pubmed, Mediline, Web of Science, Embase, Chinese Biomedical Literature Database (CBM), China National Knowledge Infrastructure (CNKI), Chinese Science Journal Database (VIP), and Wanfang Database up to June 26, 2025. The specific search strategies are shown in Supplementary material 1. There were no language restrictions. The retrieved literature was manually screened to identify potentially eligible studies.

### 2.1 Inclusion criteria

#### 2.1.1 Research design

Included in publicly published RCTs. There are no restrictions on the language of publication, country, time or trial stage.

#### 2.1.2 Research subjects

Inclusion criteria:

Type 2 diabetes mellitus (T2DM): meets the diagnostic criteria of the “Chinese Guidelines for the Prevention and T2DM (2020 Edition)”. DKD: meets the diagnostic criteria of the “Clinical Guidelines for the Prevention and Treatment of Diabetic Kidney Diseases in China”. Hyperuricemia (HUA): diagnosed based on relevant clinical diagnostic criteria. Selected patients: clearly diagnosed with DKD combined with HUA(DKD+HUA). Age: no limit. Gender and race: no restrictions. Disease staging: The DKD staging is limited to 3–4 stages and eGFR≥30 mL·min^−1^·(1.73 m^2^)^−1^.

#### 2.1.3 Intervention measures

Treatment group: On the basis of conventional basic treatment, combined with oral Febuxostat at a dose of 40 mg/day for a course of 6 months.

Control group: Only received the same conventional basic treatment as the treatment group, with a treatment course of 6 months.

#### 2.1.4 Outcome indicators

Main indicators: Total effective rate, incidence of adverse reactions.

Secondary indicators: Serum uric acid (SUA), urine albumin-to-creatinine ratio (UACR), serum creatinine (Scr), estimated glomerular filtration rate (eGFR), blood urea nitrogen (BUN).

### 2.2 Exclusion criteria

RCTs that meet any of the following conditions will be excluded: ① The study design does not conform to the RCT criteria or the clinical efficacy evaluation criteria. ② Repeatedly published research. ③ Intervention measures include traditional Chinese medicine, acupuncture or other drugs used in combination with febuxostat (only for the comparison of basic treatment + febuxostat vs. basic treatment). ④ No relevant outcome measures of concern in this study were reported. ⑤ The full text cannot be obtained. ⑥ The data contains obvious errors, omissions or incompleteness.

### 2.3 Search strategy

Databases: A systematic search was conducted in PubMed, Mediline, Embase, Web of Science, Sinomed, China National Knowledge Infrastructure (CNKI), WANFANG DATA, and VIP. The search spanned from the inception of each database to June 26, 2025. There were no restrictions on the year of publication or language.

Search Terms: The search strategy combined subject headings (MeSH/Emtree) with free-text terms. Core search terms included: Diabetic Nephropathies, Diabetic Kidney Disease, Hyperuricemia, Gout, Febuxostat, Uloric (the trade name of Febuxostat, randomized controlled trial, etc.). The search queries were constructed using Boolean logic operators (AND, OR, NOT) to combine these terms.

### 2.4 Data extraction

Literature Management: EndNote X21 (Clarivate Analytics, USA) was used to manage the search results and remove duplicate articles. Screening Process: Two researchers (Minghao Lin and Hui Zhang) independently screened the literature. Initially, articles were excluded based on titles and abstracts if they were clearly irrelevant (such as reviews, animal experiments, non-RCT studies). Subsequently, the full texts of the remaining articles were obtained and read to determine the final included studies. Disagreements during the screening process were resolved through discussion or consultation with a third researcher (Yujuan Fu). Data Extraction: A standardized data extraction form was designed using Microsoft Excel (Microsoft, USA). The two aforementioned researchers independently extracted the following information from the included studies.

Basic Study Information: First author, year of publication. Characteristics of Study Participants: Sample size of each group, gender distribution, mean age/age range, disease duration. Details of Interventions: Specific treatment regimens for the treatment and control groups (medication, dosage, duration). Outcome Data: Overall efficacy rate (the criteria for judging the efficacy of DKD referred to “The Standardization of Syndrome Differentiation and Efficacy Evaluation Scheme for Diabetic Nephropathy and Its Research”. Significant effect: UACR decreased by ≥50% compared to before treatment; Effective: UACR decreased by 30% to < 50% compared to before treatment; Ineffective: UACR did not meet the above standards or increased instead). Incidence of adverse reactions. Serum uric acid (SUA). Urinary albumin-to-creatinine ratio (UACR). Serum creatinine (Scr). Estimated glomerular filtration rate (eGFR). Blood urea nitrogen (BUN).

Disagreement Resolution: Inconsistencies during the data extraction process were resolved through double-checking the original articles or consulting a third researcher to reach a consensus.

### 2.5 Quality assessment of literature

The risk of bias in the included studies was assessed using the Cochrane Risk of Bias 2.0 tool (RoB 2.0). The assessment covered the following core domains: random sequence generation (selection bias); allocation concealment (selection bias); blinding of participants and personnel (performance bias); blinding of outcome assessment (detection bias); completeness of outcome data (attrition bias); selective reporting (reporting bias). Other potential sources of bias: each study was independently rated as “low risk,” “high risk,” or “unclear risk.” Two researchers (Minghao Lin and Hui Zhang) independently completed the assessment, with disagreements resolved through arbitration by a third researcher (Yujuan Fu).

### 2.6 Assessment of heterogeneity

Inter-study heterogeneity was quantified using the I^2^ statistic and the Cochrane Q test (significance level α = 0.10): low heterogeneity (fixed-effect model applicable): I^2^ ≤ 50% and *P* > 0.10; high heterogeneity (random-effects model applicable): I^2^ > 50% or *P* ≤ 0.10.

### 2.7 Statistical analysis

All analyses were conducted using RevMan 5.4 (Cochrane Collaboration). For dichotomous variables (such as overall efficacy rate and incidence of adverse reactions), the risk ratio (RR) and 95% confidence interval (CI) were calculated. For continuous variables (such as SUA and Scr), the weighted mean difference (WMD) or standardized mean difference (SMD) and 95% CI were calculated (depending on the consistency of the measurement scales). The combined effect size was tested using the Z-test (P < 0.05 was considered statistically significant). The model selection was based on the heterogeneity results from Section 2.6, choosing either a fixed-effect or random-effects model.

### 2.8 Assessment of publication bias

When the number of included studies was ≥10: funnel plots were used for visual assessment of small-study effects. If significant bias was detected, the trim-and-fill method was used to correct the combined effect size.

## 3 Meta-analysis results

### 3.1 Literature search

**Figure 1:**
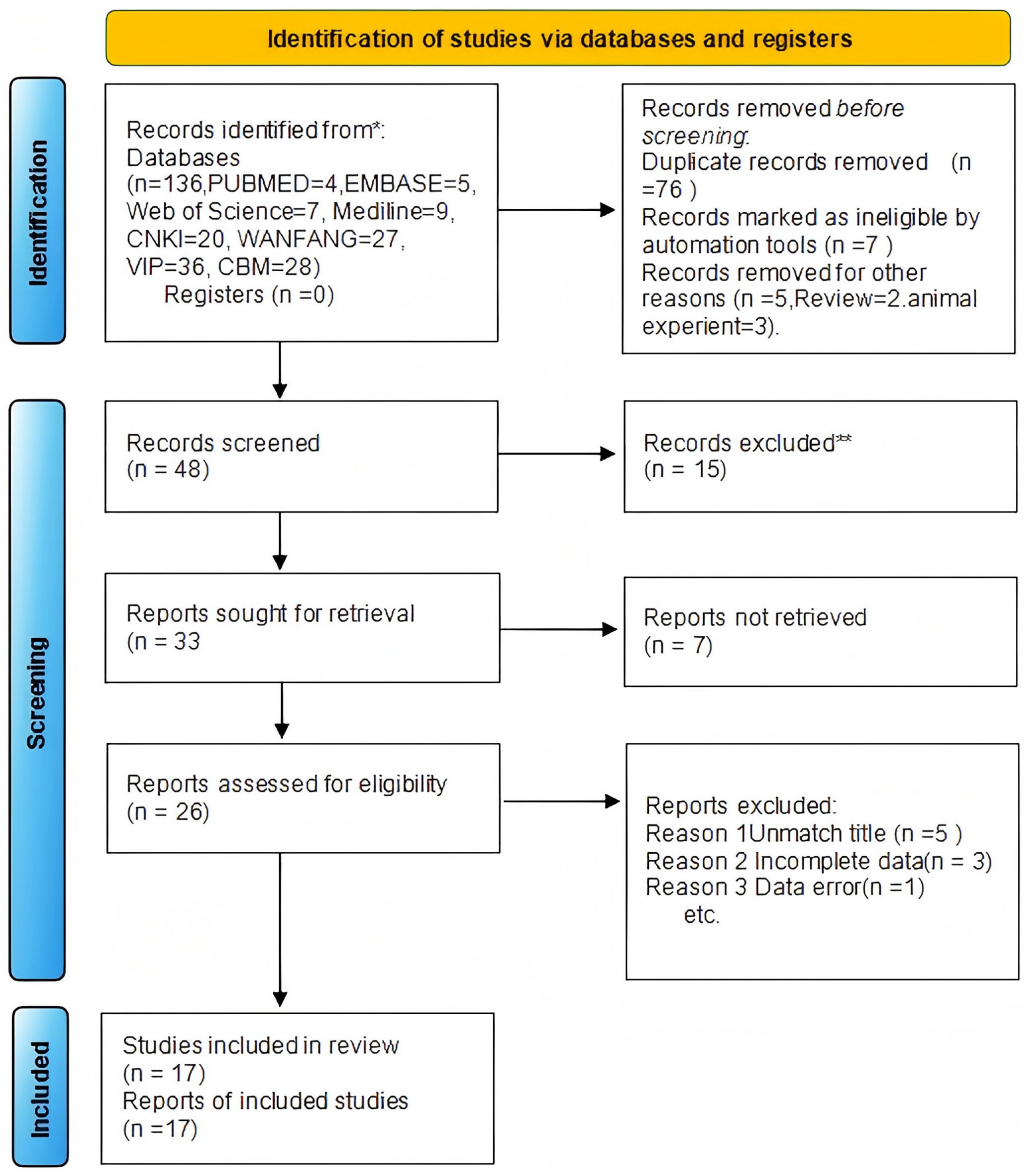
Flow diagram.

A total of 17 RCTs (11–26) were finally included (total sample size *n* = 1,300; 647 in the treatment group and 653 in the control group). All studies used febuxostat as the intervention drug. The literature search process is shown in Figure 1, and the basic information of the included studies is presented in Table 1.

**Table 1:** Basic study information.

**ID**	**Author**	**Sample size**		**Sex (M/F)**		**Age (ẋ** ±**s)**		**Age range**		**Time (ẋ** ±**s)Year**		**Intervention measure**		**dosage/per**	**Course**
		**T**	**C**	**T**	**C**	**T**	**C**	**T**	**C**	**T**	**C**	**T**	**C**	**T**	
1	Wei Qiaoyan 2021	32	32	15/17	14/18	52.9 ± 8.5	52.4 ± 8.4	-	-	7.4 ± 2.3	7.8 ± 2.5	RT+FEB	RT	40 mg	24w
2	Ma Yanlu 2023	30	30	16/14	15/15	46.1 ± 6.0	45.2 ± 6.0	29–64	30–63	-	-	RT+FEB	RT	40 mg	24w
3	Liu Dan 2018	30	30	18/12	17/13	46.3 ± 8.4	49.6 ± 7.8	–	–	5.8 ± 3.2	5.2 ± 2.4	RT+FEB	RT	40 mg	24w
4	Li Yanli 2020	40	40	25/15	29/11	55.10 ± 0.18	52.12 ± 0.21	32–72	23–75	-	-	RT+FEB	RT	40mg	24w
5	Zhu Qizhi 2022	30	30	17/13	16/14	56.2 ± 9.6	56.1 ± 9.5	35–66	34–65	-	-	RT+FEB	RT	40 mg	24w
6	Miao Yan 2019	54	58	29/25	29/29	57.2 ± 10.2	56.3 ± 9.7	32–70	34–67	8.6 ± 1.8	9.1 ± 1.5	RT+FEB	RT	40 mg	24w
7	Huang Wen 2020	18	20	16/2	17/3	58.73 ± 11.50	-	–	–	11.78 ± 5.71	12.10 ± 5.69	RT+FEB	RT	40 mg	24w
8	Sun Xin 2019	16	16	9/7	10/6	51.3 ± 10.5	51.4 ± 10.6	36–69	37–70	2.5 ± 1.3	2.4 ± 1.1	RT+FEB	RT	40 mg	24w
9	Sun Yanchun 2023	100	100	52/48	54/46	54.95 ± 1.68	54.37 ± 1.46	46–65	45–63	4.09 ± 1.15	4.37 ± 1.28	RT+FEB	RT	40 mg	24w
10	Wei Beibei 2020	45	45	24/21	23/22	49.89 ± 7.58	49.67 ± 7.65	35–74	33–72	9.12 ± 1.41	8.62 ± 1.34	RT+FEB	RT	40 mg	24w
11	Meng Xiangxue 2020	48	48	44/4	46/2	64.56 ± 3.70	64.40 ± 3.71	–	–	7.19 ± 1.48	7.15 ± 1.29	RT+FEB	RT	40 mg	24w
12	Zhao Haitao 2022	40	40	18/22	17/23	57.68 ± 6.87	57.57 ± 5.74	32–71	32–72	16.34 ± 6.37	16.46 ± 6.51	RT+FEB	RT	40 mg	24w
13	Zhong Zhenhui 2019	25	25	16/9	15/10	54.0 ± 15.0	56.5 ± 15.5	39–72	41–72	5.5 ± 2.5	6.0 ± 2.0	RT+FEB	RT	40 mg	24w
14	Zuo Weihui 2022	45	45	26/19	25/20	43.64 ± 5.02	43.23 ± 5.63	42–70	43–68	5.41 ± 1.72	5.28 ± 1.69	RT+FEB	RT	40 mg	24w
15	Fang Zhiqun 2020	25	25	15/10	17/8	48.02 ± 11.33	47.52 ± 12.31	24–84	23–81	-	-	RT+FEB	RT	40 mg	24w
16	Jing Xiaona 2020	44	44	22/22	21/23	56.51 ± 5.35	55.50 ± 5.40	42–71	41–70	9.01 ± 2.05	8.01 ± 2.01	RT+FEB	RT	40 mg	24w
17	Ding Zhengqing 2019	25	25	15/10	14/11	54.2 ± 3.58	53.4 ± 3.65	44–67	42–49	-	-	RT+FEB	RT	40 mg	24w

There were 17 studies that were included in this meta-analysis.

T, Treatment (group); C, Control (group); RT, Routine treatment (group); FEB, Febuxostat (group).

### 3.2 Quality assessment of literature

**Figure 2:**
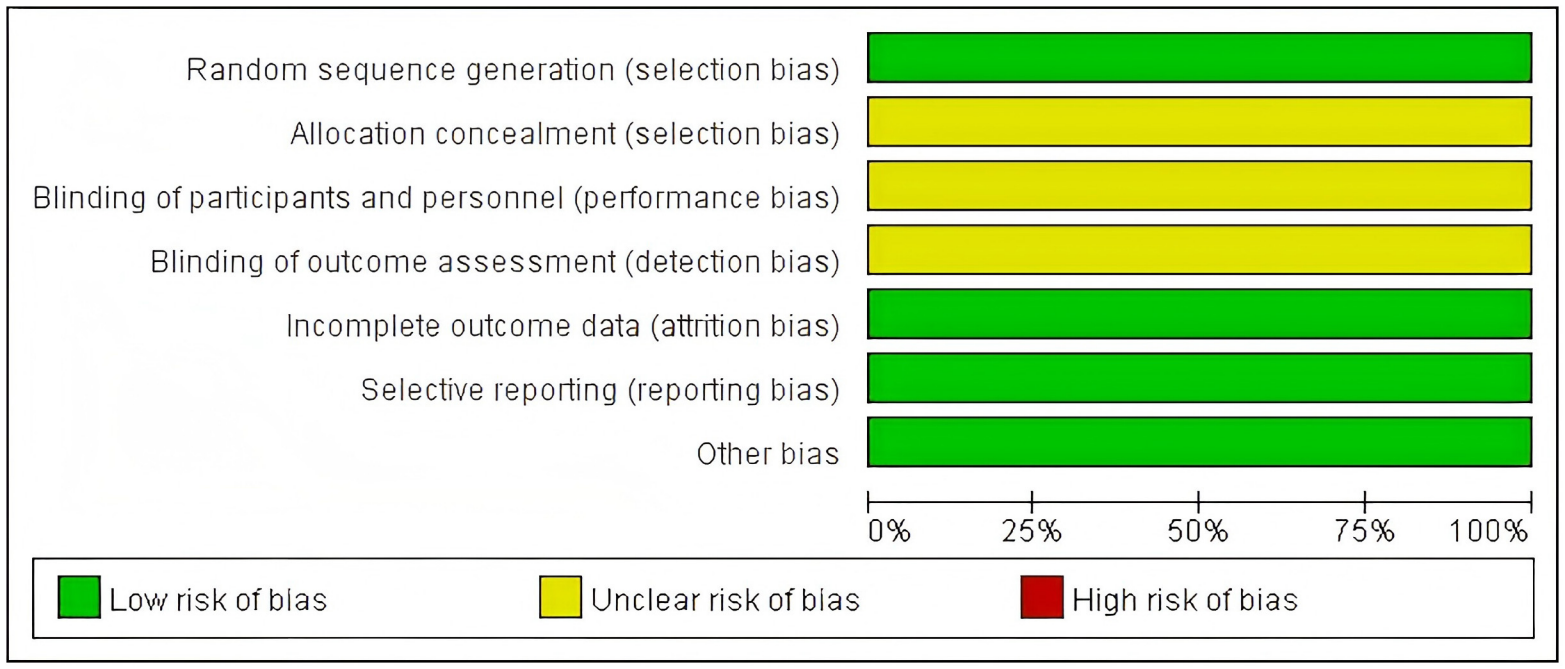
Risk of bias graph.

**Figure 3:**
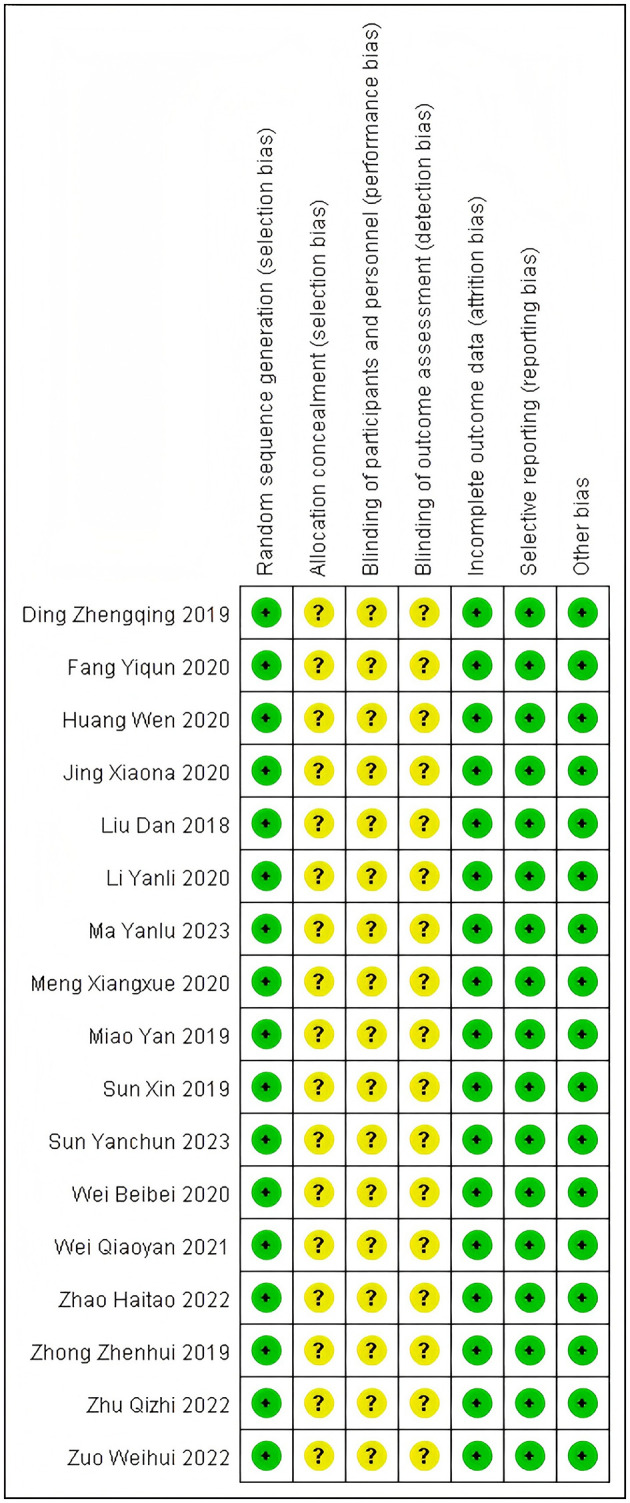
Risk of bias summary.

Assessed using the Cochrane RoB 2.0 tool. Random sequence generation: All 17 studies used a random number table method (low risk); Allocation concealment: Only 1 study explicitly described the method (low risk), while the rest did not mention it (unclear risk); Blinding of outcome assessors: None of the studies reported this (unclear risk); Completeness of outcome data: 12 studies had no dropouts/withdrawals (low risk); Selective reporting: 12 studies fully reported the pre-specified outcomes (low risk); Other biases: 12 studies did not explicitly state (low risk). As is shown in Figures 2, 3.

### 3.3 Meta-analysis

#### 3.3.1 Heterogeneity analysis

**Figure 4:**
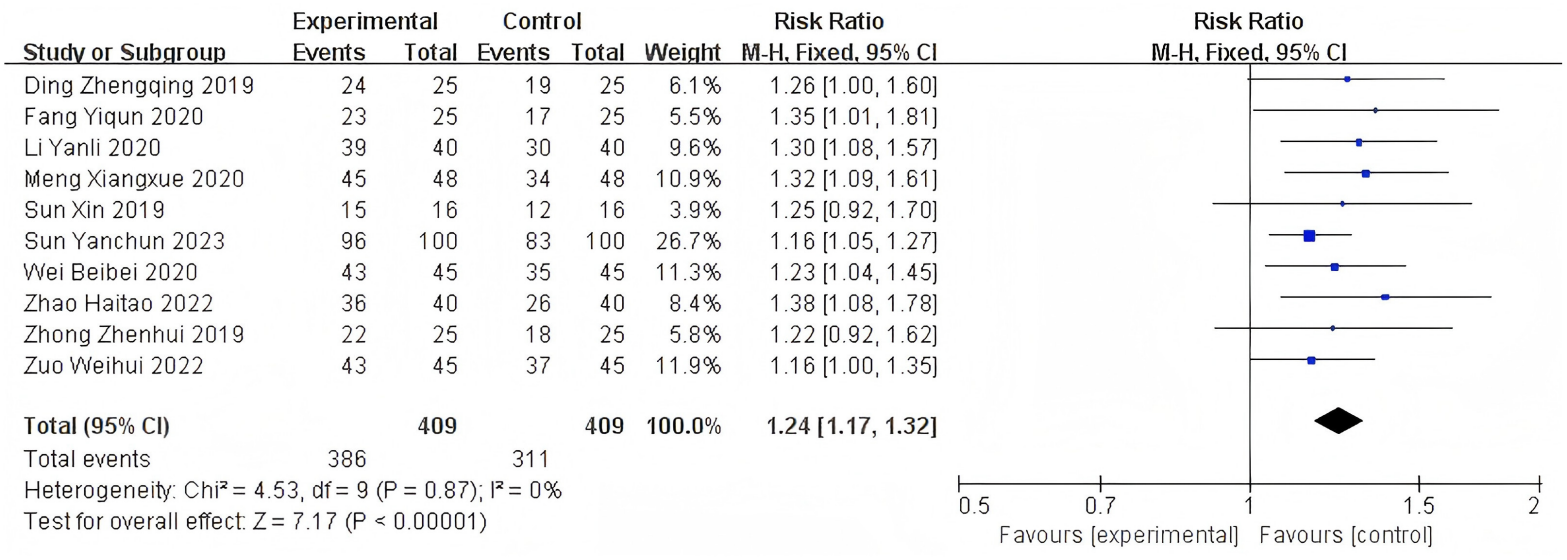
Forest plot of efficacy rate.

**Figure 5:**
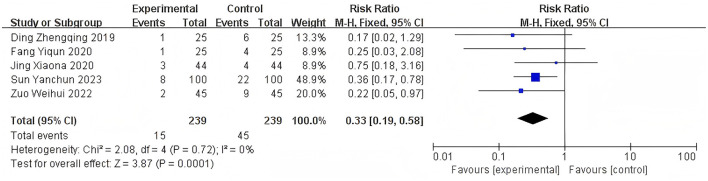
Forest plot of adverse reactions.

**Figure 6:**
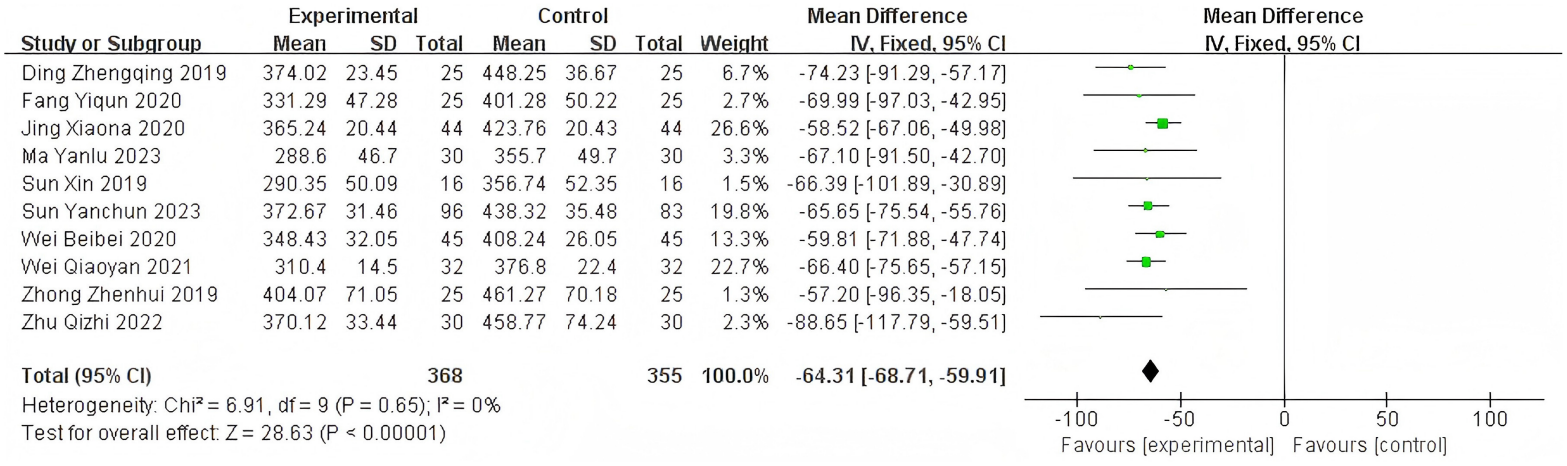
Forest plot of SUA.

**Figure 7:**
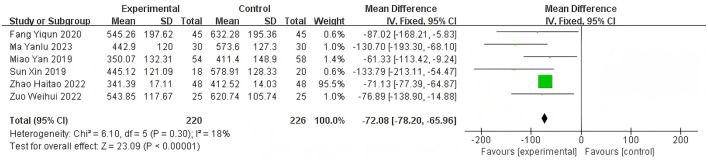
Forest plot of UACR.

**Figure 8:**
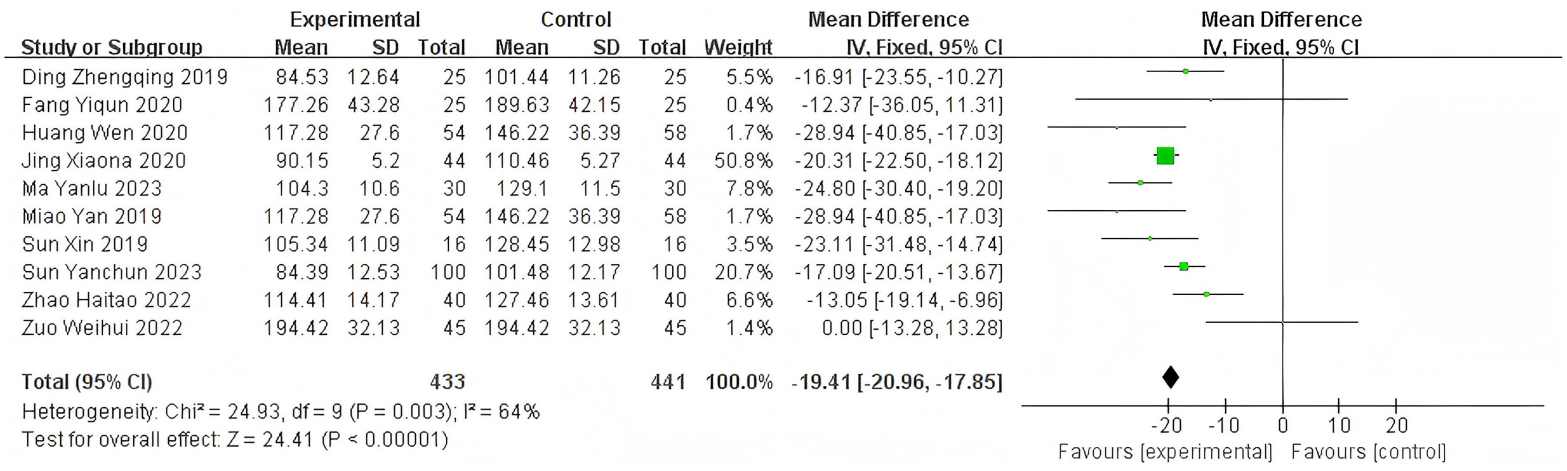
Forest plot of Scr.

**Figure 9:**
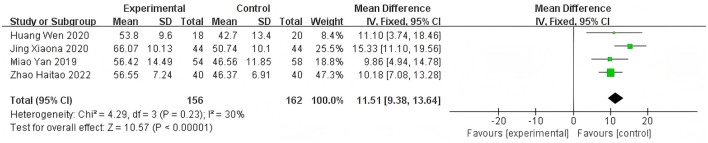
Funnel plot of eGFR.

**Figure 10:**
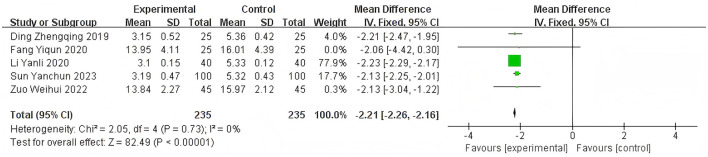
Funnel plot of BUN.

Heterogeneity of efficacy rate was tested among the ten selected studies. The test results showed that the I^2^statistic was 0%, and the *P*-value of the Q test was 1.00, which is significantly higher than the threshold of 0.1. This indicates that there was no significant statistical heterogeneity among the selected studies. Therefore, it was appropriate to use a fixed-effect model for analysis. The combined results of these ten studies showed a RR=1.24, with 95%CI [1.17, 1.32], which was statistically significant. The Z statistic was 7.17, with a *P*-value of 0.00001, < 0.05, indicating that febuxostat treatment for DKD+HUA was significantly more effective than the control group. For detailed information, please refer to the forest plot in the Appendix. For detailed information, please refer to the forest plot in the Appendix (Figure 4). Heterogeneity of adverse reactions was assessed among the five selected studies. The results indicated that the I^2^statistic was 0%, and the *P*-value of the Q test was 0.69. This suggests that there was no significant statistical heterogeneity among the included studies. Therefore, it was appropriate to use a fixed-effect model for analysis. The combined data from these five studies yielded a summary RR=0.33, with 95% CI[0.19,0.58], which was statistically significant. The specific manifestations of adverse reactions in the studies included rash, diarrhea, fever, renal function abnormalities, and liver function abnormalities. For detailed information, please refer to the accompanying forest plot. For detailed information, please refer to the forest plot in the Appendix (Figure 5). A comprehensive assessment of SUA was conducted across 12 articles. During the heterogeneity analysis, the I^2^value was as high as 80%, significantly exceeding the critical value of 50%, and the *p*-value of the Q test was < 0.01. This indicates that there was significant statistical heterogeneity among the included studies. Given this, it is particularly necessary to further explore the sources of heterogeneity. Through sensitivity analysis, we found that the studies Zuo et al. (15) and Wen et al. (11) contributed significantly to the heterogeneity. After excluding these two studies, the heterogeneity among the remaining 10 articles was significantly reduced, with an I^2^value of 0% and a *p*-value of 0.65, which exceeded the significance level of 0.1. Based on this result, this study decided to use a fixed-effect model for the meta-analysis. The combined effect size from these 10 studies was MD = −64.31, with a 95% CI [−68.71, −59.91], which was statistically significant (Z = 28.63, *P* < 0.00001). For detailed information, please refer to the forest plot in the Appendix (Figure 6). A comprehensive assessment of the UACR was conducted across seven studies. During the heterogeneity analysis, the I^2^statistic reached 83%, significantly exceeding the critical value of 50%, and the *p*-value of the Q test was < 0.01, indicating significant statistical heterogeneity among the included studies. Given this, it is necessary to further explore the sources of heterogeneity. Through sensitivity analysis, we found that the study published by Meng et al. (25) in 2020 contributed significantly to the heterogeneity. After excluding this study, the heterogeneity analysis of the remaining 6 studies showed that the I^2^value decreased to 18%, with a *p*-value of 0.30, which is higher than the significance level of 0.1. Based on this result, this study decided to use a fixed-effect model for the meta-analysis. The combined data from these 6 studies yielded a MD = −72.08, with a 95%CI [−78.20, −65.96], which was statistically significant. The Z value was 23.09, with *P* < 0.00001, indicating that febuxostat treatment for DKD+HUA was more effective in reducing UACR than the control group. For detailed information, please refer to the forest plot in the Appendix (Figure 7). A comprehensive assessment of Scr levels was conducted across 10 articles. During the heterogeneity analysis, the I^2^statistic was 64%, indicating moderate heterogeneity among the studies. Therefore, a random-effects model was chosen for the meta-analysis. The combined data from these 10 studies yielded a MD = −19.24, with a 95%CI [−20.87, −17.61], which was statistically significant (Z = 23.07, *P* < 0.00001). This indicates that febuxostat treatment for DKD+HUA is more effective in reducing Scr levels compared to the control group. For detailed information, please refer to the forest plot in the Appendix (Figure 8). An assessment of the eGFR data was conducted across the four included studies. During the heterogeneity analysis, the I^2^statistic was 30%, indicating low heterogeneity among the studies. Therefore, a fixed-effect model was chosen for the meta-analysis. The combined data from these 4 studies yielded a MD = 11.51, with a 95% CI [9.38, 13.64], which was statistically significant. The Z value was 10.57, with a *P* < 0.00001, indicating that febuxostat treatment for DKD+HUA is significantly more effective in improving eGFR compared to the control group. For detailed information, please refer to the forest plot in the Appendix (Figure 9). A comprehensive assessment of BUN levels was conducted across five relevant articles. During the heterogeneity analysis, the I^2^statistic was 0%, indicating homogeneity among the included studies. Therefore, a fixed-effect model was chosen for the meta-analysis. The combined data from these five studies yielded a MD = −2.21, with a 95% CI [−2.26, −2.16], which was statistically significant. The Z value was 82.49, with a *P* < 0.00001, indicating that febuxostat treatment for DKD+HUA is significantly more effective in reducing BUN levels compared to the control group. For detailed information, please refer to the forest plot in the Appendix (Figure 10).

#### 3.3.2 Assessment of publication bias

**Figure 11:**
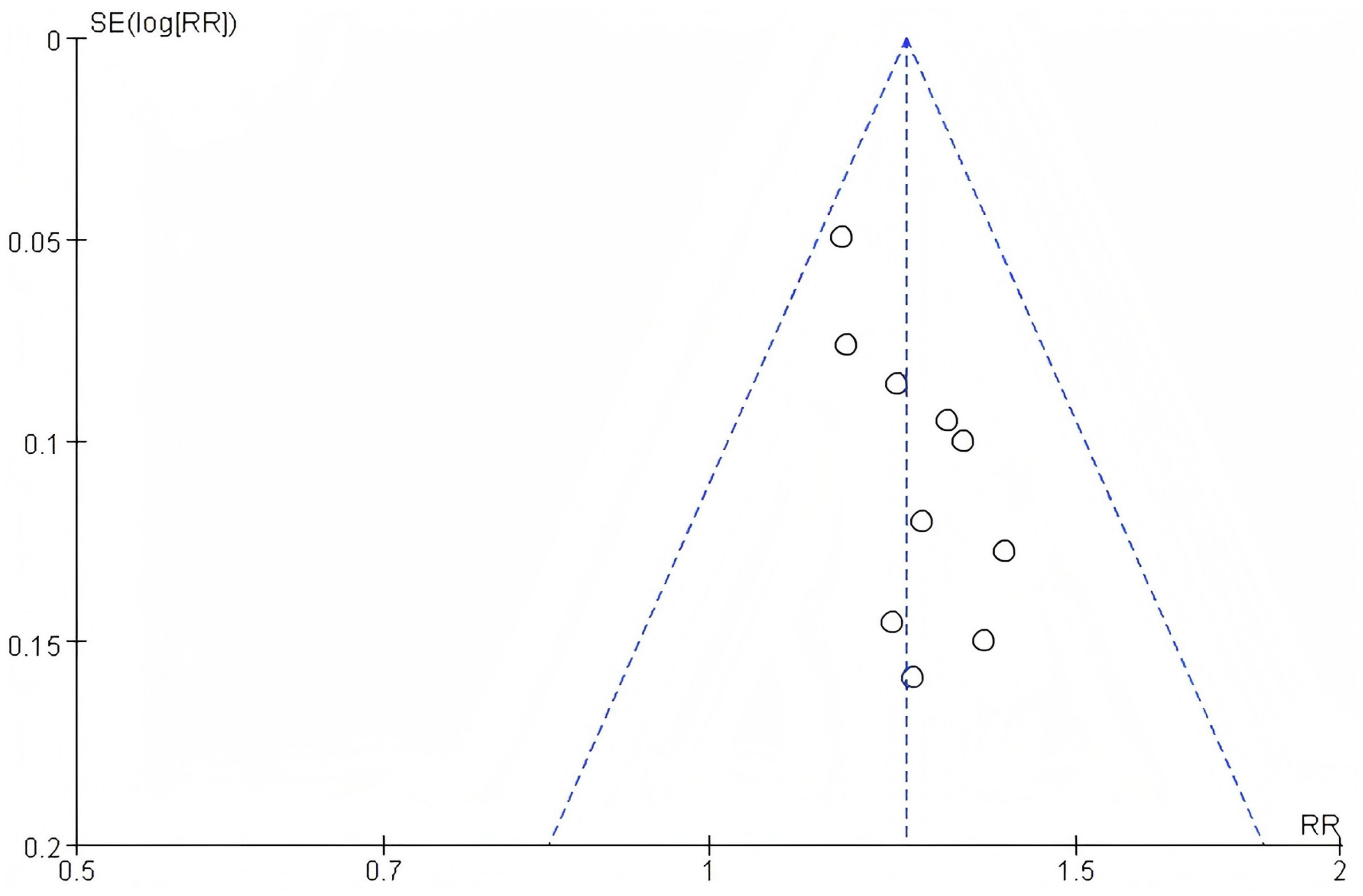
Funnel plot of efficacy rate.

**Figure 12:**
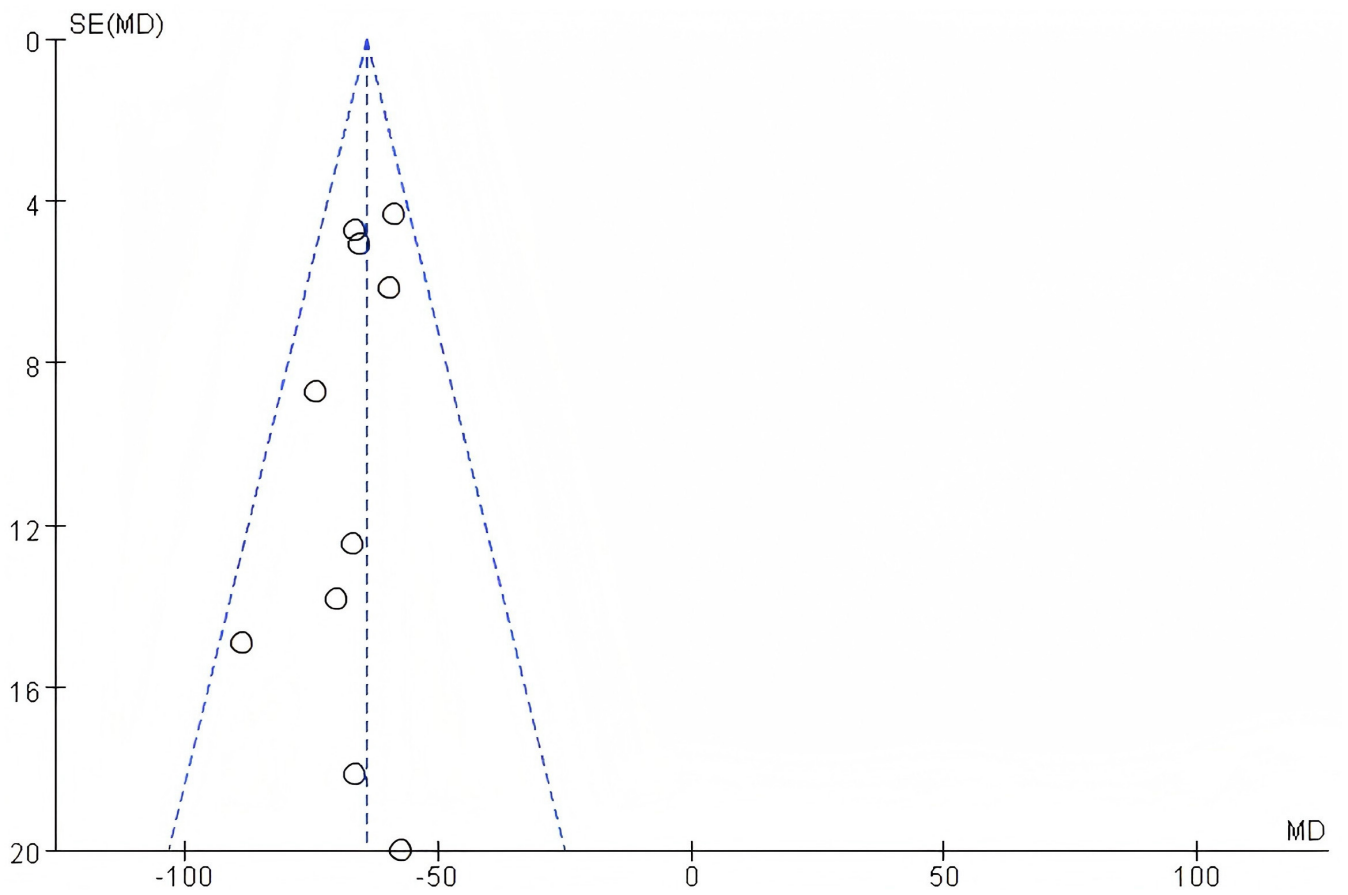
Forest plot of SUA.

**Figure 13:**
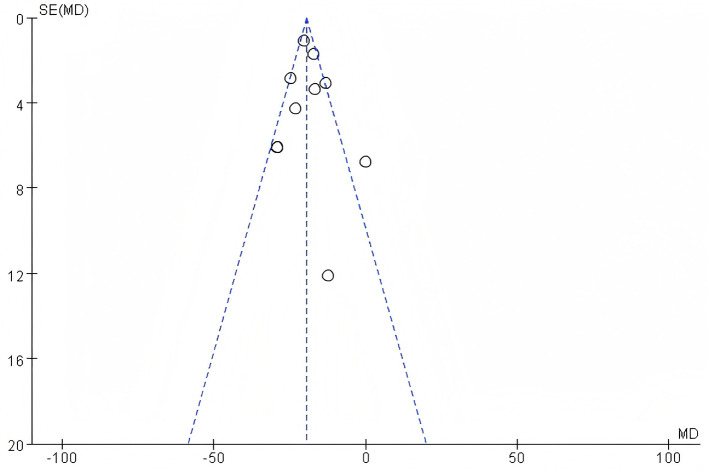
Funnel plot of Scr.

To assess potential publication bias in this study, a funnel plot of efficacy Rate was used as an analytical tool. The symmetry of the funnel plot is a key indicator for judging the presence of publication bias. By observing the funnel plot presented, it can be clearly seen that the included studies in this research show good symmetry on the funnel plot. Based on this, it can be concluded that there is no publication bias in the literature of this study. For detailed information, please refer to the funnel plot in the Appendix (Figure 11). Observing the funnel plot of SUA, it can be seen that it basically shows a symmetrical distribution. Based on this, it can be inferred that there is no publication bias in the included studies of this research. For detailed information, please refer to the funnel plot in the Appendix (Figure 12). By observing the funnel plot of Scr shown in the figure below, it can be seen that it basically shows a symmetrical distribution. Based on this, it can be judged that there is no significant publication bias in the included studies of this research. For detailed information, please refer to the funnel plot in the Appendix (Figure 13).

## 4 Discussion

This systematic review and meta-analysis integrated 17 randomized controlled trials involving 1,300 patients with hyperuricemia and diabetic kidney disease to explore the impact of febuxostat on this patient population. The results demonstrated that febuxostat significantly reduced serum uric acid levels and improved renal function-related indicators, such as lowering serum creatinine and increasing the eGFR, while also reducing proteinuria. These findings indicate that febuxostat plays a significant role in renal protection in the treatment of hyperuricemia and diabetic kidney disease, offering a new option and robust evidence-based support for clinical therapy. Compared with previous studies, this review not only focused on the control of serum uric acid by febuxostat but also emphasized the assessment of its impact on key indicators of diabetic kidney disease, such as renal function and proteinuria. Previous research has mostly concentrated on patients with gout or isolated hyperuricemia, with relatively few studies on the specific population of hyperuricemia combined with diabetic kidney disease. This review fills this gap, providing clinicians with more targeted references when treating patients with hyperuricemia and diabetic kidney disease.

Hyperuricemia is a common comorbidity of diabetic kidney disease, and the two conditions interact with each other to form a vicious cycle (27). Hyperuricemia can lead to renal damage through various mechanisms, such as the deposition of urate crystals, oxidative stress, and inflammatory responses, thereby accelerating the progression of DKD (28). Conversely, the impaired renal excretory function in patients with diabetic kidney disease can further elevate serum uric acid levels. Therefore, effectively controlling serum uric acid levels is crucial for slowing the progression of diabetic kidney disease.

At present, the commonly used uric acid–lowering drugs in clinical practice include allopurinol and benzbromarone. However, the application of these drugs in patients with hyperuricemia and diabetic kidney disease has certain limitations. Allopurinol may cause severe allergic reactions, especially in Asian populations; benzbromarone may increase the renal burden and is restricted for use in patients with renal insufficiency. Febuxostat, as a novel uric acid–lowering drug, reduces serum uric acid levels by inhibiting the activity of xanthine oxidase and has good safety and tolerability (29). However, there are currently few studies on the application of febuxostat in patients with hyperuricemia and diabetic kidney disease, and its exact efficacy and safety still need to be further clarified (30).

Based on the above background, the results of this paper have important clinical significance. Febuxostat reduces uric acid production from the source by precisely inhibiting xanthine oxidase, significantly lowering the level of SUA (31). This effect directly alleviates the multiple damages of hyperuricemia to the kidneys: ① It reduces the deposition of urate crystals, alleviates the inflammatory response and oxidative stress of the kidneys, thereby reducing UACR (32); ② Repair the endothelial function of renal vessels, improve renal blood flow and microcirculation, and increase eGFR; ③ Inhibit the process of renal interstitial fibrosis, reduce the metabolic burden on the kidneys, and promote the excretion of Scr and BUN. In patients with diabetic nephropathy complicated with hyperuricemia, febuxostat can not only effectively control uric acid and delay the progression of kidney disease, but also help restore or stabilize renal function through multiple pathways of kidney protection (anti-inflammation, anti-fibrosis, improved blood flow and antioxidation) (33). The total effective rate of its treatment is significant, which can reduce the risk of gout attacks and improve the quality of life and survival rate of patients. In addition, the drug has good safety. When used properly, the risks of liver, kidney and cardiovascular diseases are controllable, which is conducive to patients' long-term adherence to treatment and is a key choice for comprehensive management. This provides clinicians with a new and more effective treatment option when treating patients with hyperuricemia and diabetic nephropathy. Furthermore, the results of this paper also provide a direction for future clinical research and drug development, which is conducive to further exploring the application value of febuxostat in this field. This study has certain limitations. For instance, the follow-up duration of some included studies is limited, which may fail to capture very long-term cardiovascular events. Additionally, due to the constraints of original data, a more in-depth subgroup analysis based on whether patients have comorbid heart failure could not be conducted. However, studies have confirmed that febuxostat even exhibits cardioprotective and cerebroprotective effects in patients with comorbid diabetes and chronic kidney disease (34). The current controversy regarding its cardiovascular safety may stem from the heterogeneity of study populations; for example, in patients with chronic heart failure, this drug has been shown to pose potential risks (35). Furthermore, the dosage of the drug is also a key factor. The latest evidence indicates that low-dose febuxostat can not only effectively lower uric acid but also improve vascular function with good safety (36). Therefore, the cardioprotective and cerebroprotective effects of febuxostat should be comprehensively evaluated based on patients' specific comorbidities (such as whether they have comorbid heart failure) and the treatment dosage.

## 5 Conclusion

This thesis, through systematic review and meta-analysis, addresses the efficacy and safety of febuxostat in patients with hyperuricemia and diabetic kidney disease. The results indicate that febuxostat significantly reduces serum uric acid levels, improves renal function, and decreases proteinuria, demonstrating substantial renal–protective effects. Despite certain limitations, this study provides clinicians with important reference information when treating patients with hyperuricemia and diabetic kidney disease, aiding in guiding clinical practice and enhancing therapeutic outcomes. Future research should focus on conducting large-scale, high-quality clinical studies to clarify the long-term efficacy and safety of febuxostat, thereby offering more reliable evidence for the treatment of this disease.

## 6 Limitation

Although this study comprehensively assessed the application of febuxostat in hyperuricemia and diabetic kidney disease through systematic review and meta-analysis, several limitations remain. First, the sample sizes of the included studies were relatively small, and the quality of some studies could be improved, potentially introducing bias. Second, the follow-up periods of the studies were relatively short, which may not be sufficient for evaluating the long-term efficacy and safety of febuxostat. Additionally, this study was based on published literature, which may be subject to publication bias. Lastly, differences in patient baseline characteristics, treatment regimens, and evaluation indicators across studies may affect the accuracy and reliability of the results. Clearly specify the distribution ranges of eGFR included in the study and the exclusion criteria; Objectively present the combined effect size differences among different eGFR subgroups (Parazacco spilurus subsp. spilurus), highlighting the limitations of evidence for weakened protective effects when eGFR < 30; Explain the differences between this study and clinical routine medication populations (Homo sapiens) (Parazacco spilurus subsp. spilurus), as well as the necessity of conducting future RCTs targeting end-stage renal disease patients to strengthen the evidence chain. The findings of this study only reflect the impact of febuxostat on short-term surrogate markers of renal injury and cannot represent its effects on long-term renal outcomes (such as ESKD, mortality). Future large-sample RCTs with follow-up periods ≥1 year are needed, using hard endpoints as the core evaluation indicators, to further validate its clinical value.

## Data Availability

The original contributions presented in the study are included in the article/Supplementary material, further inquiries can be directed to the corresponding authors.

## References

[1] Gohda T Yanagisawa N Murakoshi M Ueda S Nishizaki Y Nojiri S . Association between kidney function decline and baseline TNFR levels or change ratio in TNFR by febuxostat chiefly in non-diabetic CKD patients with asymptomatic hyperuricemia. Front Med. (2021) 8:634932. 10.3389/fmed.2021.634932 34322499 PMC8310915

[2] Nakayama S Satoh M Toyama M Hashimoto H Murakami T Hirose T . Comparison of the incidence of proteinuria and changes in eGFR among febuxostat and topiroxostat users. Clin Exp Nephrol. (2025) 29:797–806. 10.1007/s10157-025-02630-x 39881083 PMC12125080

[3] Becker MA MacDonald PA Hunt BJ Jackson RL. Diabetes and gout: efficacy and safety of febuxostat and allopurinol. Diabetes Obes Metab. (2013) 15:1049–55. 10.1111/dom.12135 23683134 PMC3902994

[4] Nakamura T Murase T Nampei M Morimoto N Ashizawa N Iwanaga T . Effects of topiroxostat and febuxostat on urinary albumin excretion and plasma xanthine oxidoreductase activity in db/db mice. Eur J Pharmacol. (2016) 780:224–31. 10.1016/j.ejphar.2016.03.055 27038523

[5] Komers R Xu B Schneider J Oyama TT. Effects of xanthine oxidase inhibition with febuxostat on the development of nephropathy in experimental type 2 diabetes. Br J Pharmacol. (2016) 173:2573–88. 10.1111/bph.13527 27238746 PMC4978156

[6] Osonoi T Saito M Hosoya M Douguchi S Ofuchi K Katoh M. Efficacy and safety of switching from febuxostat to dotinurad, a novel selective urate reabsorption inhibitor, in hyperuricemic patients with type 2 diabetic kidney disease: Protocol for a single-arm, open-label, prospective, exploratory study. Front Endocrinol. (2022). 13:1042061. 10.3389/fendo.2022.1042061 36714585 PMC9875127

[7] Lee HJ Jeong KH Kim YG Moon JY Lee SH Ihm CG . Febuxostat ameliorates diabetic renal injury in a streptozotocin-induced diabetic rat model. Am J Nephrol. (2014) 40:56–63. 10.1159/000363421 25034030

[8] Ran J Xu G Ma H Xu H Liu Y Tan R . Febuxostat attenuates renal damage besides exerting hypouricemic effect in streptozotocin-induced diabetic rats. Int J Nephrol. (2017) 2017:2739539. 10.1155/2017/2739539 28503330 PMC5414495

[9] Fried LF Emanuele N Zhang JH Brophy M Conner TA Duckworth W . Investigators. Combined angiotensin inhibition for the treatment of diabetic nephropathy. N Engl J Med. (2013) 369:1892–903. 10.1056/NEJMoa1303154 24206457

[10] Yang KJ Choi WJ Chang YK Park CW Kim SY Hong YA. Inhibition of xanthine oxidase protects against diabetic kidney disease through the amelioration of oxidative stress via VEGF/VEGFR axis and NOX-FoxO3a-eNOS signaling pathway. Int J Mol Sci. (2023) 24:3807. 10.3390/ijms24043807 36835220 PMC9961241

[11] Wen H Yongling Z Shuying Z Jiali W Yanling Z. Effect of febuxostat on renal function in patients from South China with CKD3 diabetic nephropathy. J Bras Nefrol. (2020) 42:393–9. 10.1590/2175-8239-jbn-2019-0091 32701116 PMC7860659

[12] Wei Q Huang Z. The effects of nisoldipine on serum uric acid, urine protein excretion rate and oxidative stress in patients with early-stage nephropathy and asymptomatic hyperuricemia in type 2 diabetes. Chinese Foreign Med Res. (2021) 19:38–40. 10.14033/j.cnki.cfmr.2021.01.013

[13] Jing X Ren D Guan Q. The effect of nifedipine on renal function and inflammatory factor levels in patients with diabetic nephropathy combined with hyperuricemia. Int J Transplant Hemodial. (2020) 18:1–4.

[14] Fang Y Qiu F Huang W. Efficacy and safety of febuxostat tablets in the treatment of type 2 diabetes mellitus nephropathy with hyperuricemia. Contemp Med. (2020) 26:77–9.

[15] Zuo W. Evaluation of the advantages of Fenbixitine tablets in treating diabetic nephropathy combined with hyperuricemia. Chinese J Sci Technol Database Med. (2022) 17–20.

[16] Ding Z. The effect of febuxostat on patients with type 2 diabetes nephropathy and hyperuricemia and its effect on renal function. Big Doctor. (2019) 4:1–2.

[17] Sun X. Clinical effect of febuxostat on type 2 diabetes nephropathy combined with hyperuricemia. Chinese Med Guide. (2019) 17:47–8. 10.15912/j.cnki.gocm.2019.20.031

[18] Zhong Z Long H. Clinical efficacy and safety analysis of febuxostat in the treatment of type 2 diabetes nephropathy combined with hyperuricemia. Chinese J Tradit Western Med. (2019) 17:68–9. 10.14033/j.cnki.cfmr.2019.13.029

[19] Ma Y. Clinical effect analysis of febuxostat in the treatment of type 2 diabetic nephropathy combined with HUA. Chinese Modern Drug Appl. (2023) 17:106–8.

[20] Liu D Hongyan W. Observation on the efficacy of Fenbuxitran in treating early kidney disease combined with asymptomatic hyperuricemia in type 2 diabetes mellitus. Anhui Med. (2018) 22:1968–71.

[21] Zhu Q. The efficacy and effect on renal function of febuxostat in treating non-end-stage diabetic nephropathy with hyperuricemia. Chinese Pharm. (2022) 31:111–3.

[22] Miao Y Yan L Dong Y Zhu Q Shao F. Efficacy analysis of febuxostat in treating non-end-stage diabetic nephropathy with hyperuricemia and its effect on renal function. In: Presented at the 2018 Annual Academic Conference of the Chinese Society of Integrated Traditional and Western Medicine. Chongqing (2018).

[23] Zhao H. Clinical efficacy analysis of febuxostat in the treatment of diabetic nephropathy combined with hyperuricemia. Med Diet Health. (2022) 20:41–3, 47.

[24] Li Y Liu X Men B Cao Y Cong W. Analysis on the effectiveness of febuxostat in patients with non-end stage diabetic nephropathy combined with hyperuricemia and its effect on renal function. Famous Doctors. (2020) 2020:223.

[25] Meng X. Effects of xanthine oxidase inhibitors on vascular endothelial function in patients with type 2 diabetes nephropathy and hyperuricemia. Anhui Med J. (2020) 41:644–8.

[26] Wei B. The effects of nifedipine on renal function and prognosis in patients with early diabetic nephropathy with hyperuricemia. Mod Med Health Res. (2020) 4:37–39.

[27] Takahashi K Mizukami H Osonoi S Ogasawara S Hara Y Kudoh K ., Inhibitory effects of xanthine oxidase inhibitor, topiroxostat, on development of neuropathy in db/db mice. Neurobiol Dis. (2021) 155:105392. 10.1016/j.nbd.2021.105392 34000416

[28] Tiku A Johnson DW Badve SV. Recent evidence on the effect of urate-lowering treatment on the progression of kidney disease. Curr Opin Nephrol Hypertens. (2021) 30:346–52. 10.1097/MNH.0000000000000699 33767063

[29] Choi C Kim MG Kim JH. Reno-protective effects of xanthine oxidase inhibitors in patients with type 2 diabetes and chronic kidney disease: a systematic review and meta-analysis. J Nephrol. (2025) 38:393–401. 10.1007/s40620-024-02199-w 39865217

[30] Tian R Peng R Yang Z Peng YY Lu N. Supplementation of dietary nitrate attenuated oxidative stress and endothelial dysfunction in diabetic vasculature through inhibition of NADPH oxidase. Nitric Oxide. (2020) 96:54–63. 10.1016/j.niox.2020.01.007 31972252

[31] Mizuno Y Yamamotoya T Nakatsu Y Ueda K Matsunaga Y Inoue MK ., Xanthine oxidase inhibitor febuxostat exerts an anti-inflammatory action and protects against diabetic nephropathy development in KK-Ay obese diabetic mice. Int J Mol Sci. (2019) 20:4680. 10.3390/ijms20194680 31546603 PMC6801943

[32] Preitner F Laverriere-Loss A Metref S Da Costa A Moret C Rotman S . Urate-induced acute renal failure and chronic inflammation in liver-specific Glut9 knockout mice. Am J Physiol Renal Physiol. (2013) 305:F786–95. 10.1152/ajprenal.00083.2013 23804456

[33] White WB Saag KG Becker MA Borer JS Gorelick PB Whelton A . CARES Investigators. Cardiovascular safety of febuxostat or allopurinol in patients with gout. N Engl J Med. (2018) 378:1200–10. 10.1056/NEJMoa1710895 29527974

[34] Kang EH Choi HK Shin A Lee YJ Lee EB Song YW . Comparative cardiovascular risk of allopurinol versus febuxostat in patients with gout: a nation-wide cohort study. Rheumatology. (2019) 58:2122–9. 10.1093/rheumatology/kez189 31098635

[35] Yokota T Fukushima A Kinugawa S Okumura T Murohara T Tsutsui H. Randomized trial of effect of urate-lowering agent febuxostat in chronic heart failure patients with hyperuricemia (LEAF-CHF). Int Heart J. (2018) 59:976–82. 10.1536/ihj.17-560 30101851

[36] Mackenzie IS Ford I Nuki G Hallas J Hawkey CJ Webster J . Long-term cardiovascular safety of febuxostat compared with allopurinol in patients with gout (FAST): a multicentre, prospective, randomised, open-label, non-inferiority trial. Lancet. (2020) 396:1745–57. 10.1016/S0140-6736(20)32234-0 33181081

