# Supplementary material for: Evaluation of febuxostat in treating diabetic kidney disease with hyperuricemia: a systematic review and meta-analysis of randomized controlled trials
Source: Front Med (Lausanne). 2025 Oct 27;12:1657274. doi: 10.3389/fmed.2025.1657274 (PMC12597919; doi:10.3389/fmed.2025.1657274)
Supplement: Supplementary file 1 [file Data_Sheet_1.docx]

Supplementary material 1 Strategy of Pubmed

| Order | Strategy |
| --- | --- |
| #1 | Search: Febuxostat[Mesh] |
| #2 | Search: Diabetic Kidney Disease[Mesh] |
| #3 | Search: Diabetes nephropathy[Title/Abstract] OR Diabetic nephropathy[Title/Abstract] OR Diabetic nephrosis[Title/Abstract] OR Renal diabetes[Title/Abstract] |
| #4 | Search:#2 OR #3 |
| #5 | Search: Hyperuricemia[Mesh] |
| #6 | Search: HUA[Title/Abstract]OR Arthrolithiasis[Title/Abstract] |
| #7 | Search: #5 OR #6 |
| #8 | Search: Randomizedcontrolledtrial[Title/Abstract] OR Randomized[Title/Abstract] OR Placebo[Title/Abstract] |
| #9 | #1 AND #4 AND #7 AND #8 |
